# Role of Alendronate in Managing Osteoporosis in Celiac Disease – Illustrative Case Report

**DOI:** 10.4021/gr279w

**Published:** 2011-01-20

**Authors:** David Widjaja, Kalyan C. Kanneganti, Madanmohan Patel, Sridhar S. Chilimuri

**Affiliations:** a Department of Medicine, Bronx Lebanon Hospital Center, 1650 Selwyn Ave, 10th Floor, Bronx, NY 10457, USA

**Keywords:** Celiac disease, Osteoporosis, Alendronate

## Abstract

Management of bone density loss, as the result of calcium malabsorption in celiac disease, is critical in preventing premature bone fracture. As many of these patients need follow-up with primary care providers, internists are expected to be aware of screening and prompt management of osteopenia or osteoporosis in celiac disease. We present a case of a 32-year-old man with celiac disease who was diagnosed with osteoporosis. He was treated with calcium, vitamin D and alendronate which improved bone mineral density. This case illustrates the importance of using bisphosphonate in treating osteoporosis in celiac disease.

## Introduction

Celiac disease (CD) is defined as a small bowel disorder characterized by mucosal inflammation, villous atrophy, and crypt hyperplasia, which occurs upon exposure to gluten and demonstrates improvement with withdrawal of gluten from the diet. As a result of mucosal destruction, patients with CD may suffer from conditions caused by nutrient deficiency, including decreased bone mineral density. Untreated celiac patients may develop very low bone mineral density (BMD) or osteoporosis. Several studies suggest that restoration of bone mass in these patients is frequently incomplete, with many remaining below comparable values [1-7].

Bisphosphonates have been established as effective antiresorptive agents for the prevention and treatment of osteoporosis in postmenopausal women. Bisphosphonates are also used for treatment of Paget’s disease of bone, myeloma and bone metastases. The use of bisphosphonates for patients with CD and osteoporosis has been recommended by American Gastroenterological Association and British Society of Gastroenterology [8, 9], however currently there is no study reporting the use of bisphosphonates in CD. We present a case of a young man with CD who was then found to have asymptomatic osteoporosis. Combination therapy of gluten free diet, calcium, vitamin D, and bisphosphonate made significant improvement in bone mineral density in this patient.

## Case Report

A 32-year-old South Asian man initially presented to a gastroenterologist after abnormal liver function tests and mild anemia were detected on a routine physical examination. A colonoscopy and sonogram of the liver were performed. The colonoscopy was normal and the sonogram showed fatty liver. The patient remained asymptomatic afterwards except for occasional heartburn. At this time he was seen in our Gastroenterology (GI) clinic and further evaluation was initiated. A thorough history and physical examination were performed. The patient denied any diarrhea or history of recent travel, though his past travel history included a six-year period of stay in the Caribbean Islands five years prior to this presentation. Family history was not contributory. The patient denied any toxic habits. Physical examination was normal. With a clinical suspicion of Celiac disease laboratory work was requested which showed anti-gliadin IgA, IgG, anti-endomysial and tissue transglutaminase antibodies to be positive. Initial aminotransferases tests showed aspartate aminotransferase (AST) of 111 U/L and alanine aminotransferase (ALT) of 120 U/L. Esophagogastroduodenoscopy (EGD) was performed which showed a partial Schatzki ring in the lower third of esophagus and a diffuse granular mucosa with fissuring in the second and third part of the duodenum. Biopsy of the second part of the duodenum showed villous atrophy with crypt elongation, replacement of surface epithelial cells by cuboidal cells with loss of brush border and increase in lymphocytes relative to the number of epithelial cells. Lamina propria showed increased plasma cells, lymphocytes and polymorphs consistent with celiac disease.

Bone scan revealed osteoporosis involving lumbar spine and osteopenia involving both hips. T and age matched Z scores are shown in Table 1. The patient was started on a strict gluten free diet, vitamin D, calcium and alendronate 10 mg orally every day. Subsequent endoscopy done 6 months later showed grade I esophagitis, erythematous non-bleeding gastropathy and granular mucosa in the third part and bulb of the duodenum. Repeat biopsies of the duodenum showed regeneration of villous height and regression of crypt hyperplasia. The surface epithelium showed increased columnar cells with basal nuclei and brush borders consistent with partial restoration and improvement in celiac disease. Repeat bone scan eighteen months later showed osteopenia with significant improvement of BMD (Table 1). No significant symptoms related to side effects of alendronate were reported during the 18 months treatment period. Repeat aminotransferases revealed AST of 26 U/L and ALT of 42 U/L.

**Table 1:** Bone Mineral Density at Diagnosis and After 18 Months of Alendronate Treatment in a 32-Year-Old Patient With Osteoporosis Secondary to Celiac Disease

	Bone scan at diagnosis		Bone scan after treatment	
	T - score	Z - score	T - score	Z - score
Lumbar spine	-2.8	-2.4	-1.9	-1.6
Right hip	-1.9	-1.7	-2.0	-1.7
Left hip	-2.1	-1.9	-1.7	-1.4

## Discussion

Celiac sprue causes destruction of small bowel mucosa. The disease leads to malabsorption of multiple nutrients including folate, iron, calcium, vitamin D and protein. The destruction of proximal jejunal mucosa may impair calcium absorption, causing secondary hyperparathyroidism, increased bone resorption and excessive rates of bone loss [10-12]. As absorption of calcium is influenced by vitamin D, malabsorption of vitamin D further decreases bone mineral density in celiac sprue patients. Malabsorption of both calcium and vitamin D progresses to osteopenia and osteoporosis. Bone loss may also exist in celiac sprue in absence of gastrointestinal symptoms [13-16], therefore it is very important to perform screening for osteopenia or osteoporosis in all patients with CD. On the other hand, screening of CD may be important in young individuals who have unexplained osteopenia or osteoporosis. The severity of bone loss is also determined by gender and menopausal status [16]. Premenopausal women with CD are least likely to have osteopenia or osteoporosis compared to men or postmenopausal women with CD, therefore Dual-energy X-ray absorptiometry (DEXA) scan may reveal more significant findings in this patient population. Even, male patients were found to have lower Z-scores of lumbar spine and femoral neck than postmenopausal patients [16].

Our case of this 30 year-old-man with celiac sprue who developed osteoporosis of lumbar spine improved significantly after treatment with alendronate in addition to gluten free diet and treatment with calcium and vitamin D. His bone mineral density (BMD) had increased 10.7% over 18 months of treatment. Although gluten free diet is the corner stone of celiac sprue therapy, several studies showed that the diet alone did not improve BMD significantly [10, 16-19]. BMD may increase during the first years after institution of a gluten free diet, but most studies reveal that restoration of bone mass is frequently incomplete with many remaining below comparable control values [1-7]. The increase of BMD observed when the diet therapy is initiated is likely due to mineralization of remodeling space expanded by secondary hyperparathyroidism, therefore the BMD improves for only 2 to 3 years and then stabilizes. Remaining deficit in BMD could be then attributed to a lifelong disease which had interfered with attainment of peak bone mass [16]. Our patient was diagnosed and started on gluten free diet treatment at the age of 30 years, therefore it is plausible that he might have not achieved normal peak bone mass. Increasing BMD in this patient could be attributed to adherence with low gluten diet. However, the average rate of increase BMD was to be less than 5% in the first year of diet treatment [8].

In patients with CD, supplemental calcium and vitamin D have been recommended for prevention of secondary hyperparathyroidism by several experts [9, 20]. However, one study reported that re-mineralization in patients treated with diet and supplements was similar to that of patients treated with diet only [4]. Moreover, high serum concentration of parathyroid and 25 OH vitamin D could progress to normal level in patients treated by gluten free diet without the calcium and vitamin D supplementation [21]. Our patient was receiving calcium and vitamin D supplementation, however this supplementation was less likely to be a prime factor in the increase of BMD.

Bisphosphonates are stable analogues of pyrophosphate which inhibit bone resorption by being selectively taken up and adsorbed to mineral surfaces in bone, where they interfere with the action of osteoclasts. American Gastroenterological Association (AGA) recommends using bisphosphonate if: (i) T score -2.5 to -1 in setting of prolonged corticosteroid use; (ii) T score < -2.5; (iii) vertebral compression fractures regardless of T score. British Society of Gastroenterology recommends bisphosphonates for postmenopausal women with osteoporosis, men above 55 years of age with osteoporosis and patients with fragility fractures [8]. These expert recommendations have been based on several studies primarily established in postmenopausal women [22-27]. Alendronate treatment had been reported in several studies to be effective in improving BMD in men with osteoporosis, similar to its efficacy in women [28-32]. In all the studies, improvement of BMD was observed higher in lumbar spine as compared to femoral neck. The efficacy of alendronate in improving BMD was showed in either case of secondary osteoporosis or idiopathic osteoporosis [29, 30]. Compared to etiodronate, alendronate therapy led to significantly greater gains in BMD of lumbar spine [32]. In 1-year observation of alendronate therapy, the mean BMD improvement of lumbar spine was 5.4% - 7.0% and the mean improvement of femoral neck was 2.6% - 4.5% [28, 29]. Meanwhile in 2 years observation, the mean BMD improvement in lumbar spine was 7% and the mean improvement in femoral neck was 2% [31]. Based on these data, the 10.6% improvement of BMD in our patient after 18 months of therapy might mostly occur during the first 12 months of therapy.

Currently there is no study or report regarding using bisphosphonate in young men with osteoporosis caused by celiac disease. The significant increase of BMD in our patient after receiving alendronate is the first case report of alendronate treatment in celiac disease. Previous studies of alendronate therapy among men with secondary osteoporosis included only patients with long-term corticosteroid treatment, low serum testosterone, and malabsorption syndrome without specific diagnosis of celiac disease [29, 31-33]. Study comparing gluten free diet therapy only and gluten free diet therapy with bisphosphonate, especially in non-postmenopausal women, may reveal the efficacy of bisphosphonate in celiac disease.

In summary, osteopenia or osteoporosis should be clinically suspected by primary care physicians for all patients with CD, especially in men and postmenopausal women. Early diagnosis and prompt treatment of bone mass loss in this patient population may decrease incidence of fracture and premature disability. In our case, bisphosphonate therapy was shown to be effective in increasing BMD. Referral for follow-up EGD is needed in patients with CD to evaluate improvement of small bowel mucosa. Combination therapy of gluten free diet, calcium, vitamin D and bisphosphonate may be beneficial in improving BMD.
